# Choking under pressure: the neuropsychological mechanisms of incentive-induced performance decrements

**DOI:** 10.3389/fnbeh.2015.00019

**Published:** 2015-02-10

**Authors:** Rongjun Yu

**Affiliations:** ^1^Department of Psychology, National University of SingaporeSingapore, Singapore; ^2^Center for Life Sciences, Singapore Institute for Neurotechnology (SINAPSE), National University of SingaporeSingapore, Singapore

**Keywords:** stress, decision making, choking, attention, motivation, fMRI

## Abstract

In contrast to the assumption of efficiency wage models, which state that wage incentives should be positively correlated with productivity, high incentives may produce performance decrements in real life scenarios. Such a “choking under pressure” phenomenon exemplifies how psychological stress can profoundly shape human behavior, for good or for bad. Previous theories suggest that individual choking under pressure because that high pressure may distract individuals’ attention away from the task (the distraction account), raise the attention paid to step-by-step skill processes (the explicit monitoring account), or elevate the arousal in general (the over-arousal account). Recent neuroimaging studies have shown that several brain regions implicated in motivation and top-down control of attention also play a key role in stress-induced choking, supporting for the over-arousal and distraction theories of choking. This review aims to identify psychological factors that determine choking and the neural underpinnings of these processes. Insights into how incentives influence performance may aid engineering training regimens and interventions that equip individuals to better handle high-stakes-induced psychological stress, and to thrive under stress.

*By “guts” I mean, grace under pressure*.

*Ernest Hemingway*

## Introduction

At the 2004 Olympics in Athens, in the men’s 50 m rifle event, Mathew Emmons was one shot away from a gold medal. He fired. It was a bull’s eye, only at the wrong target.

Beijing, 2008, again, Mathew Emmons only needed a 6.7 to win gold. He put his finger on the trigger and fired. It was a 4.4, ridiculously below his standards.

With Olympic gold on the line, Mathew Emmons was definitely motivated to do well. However, surprisingly, his performance turned out to be a catastrophic failure when on the brink of securing a historic victory. High rewards or the threat of severe punishment can provide strong motivation, but can also induce performance decrements due to the high psychological pressure. Such a high rewards-induced performance decrements phenomenon, or so called “choking under pressure”, contradicts what efficiency wage models would predict. Efficiency wage models predict a positive relationship between wage incentives and productivity, such that a high wage produces high motivation, which, in turn, leads to better performance. However, the observation that high reward levels can have detrimental effects on performance is not uncommon in real-life. Preparing for the Olympic Games or critical academic tests (e.g., Graduate Record Examinations (GRE) or Scholastic Aptitude Test (SAT) tests) can be stressful. The excessive stress can have a profound impact on individuals’ performance. Although choking under pressure is a big concern in high performance sports and other human endeavors, the psychological and neural mechanisms underlying such a paradoxical incentive-performance relationship are not well understood yet. This review summarizes recent and accumulating evidence showing that psychological pressure can hurt performance, and discusses theoretical accounts of this phenomenon. Psychological stress, induced by the Trier Social Stress Test (TSST) or cold pressor test, can produce myriad effects on human behavior, including decision making (van den Bos et al., 2009; Starcke and Brand, 2012; Morgado et al., 2014) and memory (Schwabe and Wolf, 2013; Wingenfeld and Wolf, 2014). In the present review, I focus on the detrimental effects of incentive induced stress on economic decision making as well as cognition and sensorimotor performance. Recent studies on the neural underpinnings of these processes are also summarized and how these neuroimaging findings can be used to differentiate among alternative theories of choking are discussed. I also review behavioral interventions utilized to combat choking under pressure and point our directions for future research and practice.

## Choking via distraction

Attention is a key component in cognition and sensorimotor performance. Two prominent attentional models have been proposed to explain the detrimental effects of performance pressure. The distraction theory proposes that pressure causes a distracting environment thereby drawing performers’ attention away from skill execution (Wine, 1971; Carver and Scheier, 1981). Attentional focus is shifted to task-irrelevant cues, such as worries about the consequences. In working memory intensive tasks, such as mathematical problem solving, these mental distractions compete for and reduce working memory capacity that would otherwise be needed to perform at an optimal level. It has been demonstrated that psychological pressure harms individuals most qualified to succeed by consuming the working memory capacity that they rely on for their superior performance (Beilock, 2008). On tasks that load heavily on working memory, higher working memory individuals are most susceptible to performance decrements in stressful situations (Mattarella-Micke et al., 2011).

## Choking via explicit monitoring

On the other hand, the explicit monitoring theory, also called self-focus theory or execution focus theory, proposes that pressure increases monitoring of explicit processes by shifting mental processes from “automatic” to “controlled” (Baumeister, 1984). According to explicit monitoring theory, it is not depleted attention but unnecessarily excessive attention paid to the task execution process that causes choking. When the performers are at the novice level, they generally begin with unintegrated explicit knowledge of the task that is overtly controlled in a step-by-step manner through working memory. At this stage, individuals learn specific rules of which they are consciously aware and that they are able to verbalize. After deliberate and repeated practice, performers are able to refine and transfer explicit knowledge into implicit knowledge, which is fast, automatic and controlled without reference to working memory. The implicit knowledge entails abstract, unconscious information we know but cannot articulate effectively. Pressure raises self-consciousness and anxiety about performing correctly, which increases the attention paid to skill processes and their step-by-step control. For skills that are well-learned and proceduralised, attention to task execution at the step-by-step level disrupts the automated functioning of such skills. Thus, performers may regress to the erratic and inefficient style of execution at the beginner level. For example, research has shown that expert golfers performed putts better when their attention was drawn away from execution via secondary task demands or when emphasizing putting speed (Beilock et al., 2004a). Choking was diminished by self-consciousness training, which deals with situations that raise self-awareness and self-focusing (Beilock and Carr, 2001).

The two prominent attentional models of choking under pressure hold that pressure either distracts an individual from the task at hand (distraction theories) or causes the individual to explicitly monitor the performance of overlearned tasks in a counterproductive manner (explicit monitoring theories). These two effects are differentially relevant to performance depending on the specific nature of the task performed and the individual’s skill levels. In some circumstances, the two accounts of choking might both contribute to the deterioration in performance (Beilock et al., 2004b). For example, after the individual’s attention is switched from implicit to explicit, it would require working memory to process these explicit instructions. Because pressure also consumes working memory capacity, it will further deplete attentional resources needed to do the task in a “step-by-step” way (Gucciardi et al., 2010).

## Choking via over-arousal

Incentives are strongly linked to motivation. Thus, such incentive-induced motivation might underlie the choking processes. Another theory of choking, favored by behavioral economists, proposes that degraded performance is elicited by excessive arousal induced by high incentives or social pressure. According to the Yerkes–Dodson law (Yerkes and Dodson, 1908), which posits that there is an optimal level of arousal for executing tasks, the enhanced arousal may improve performance on simple or well-learned tasks, but impair performance on complex or not well-learned tasks. Individuals are generally motivated to convey a good image of self to others. Thus, not only can very high reward levels have a detrimental effect on performance, the mere presence of an audience can produce social pressure that hurts performance in certain circumstances. Specific to social situations, the social facilitation theory suggests that the mere or imagined presence of people in social situations creates an atmosphere of evaluation, which leads to heightened arousal (Zajonc, 1965). If the task is simple and over-learned, such as cycling, the presence of a passive audience facilitates the performance of a simple task (Zajonc, 1965). When the task is difficult and not well practiced by the individual, such as mathematical calculation, an audience inhibits the performance, possibly due to excessive psychological pressure or stress (Zajonc, 1965). In experienced basketball players, those who are high in fear of negative evaluation (FNE), measured via a brief FNE questionnaire, exhibited a significant decrease in performance when pressure went from low to high (Mesagno et al., 2012). The presence of others, either passive inattentive persons, a purportedly friendly audience, or an adversarial audience, is a sufficient condition for social facilitation and social interference effects (Butler and Baumeister, 1998; Dohmen, 2005). Moreover, the challenge and threat hypothesis further offers a biopsychosocial account for this process (Blascovich and Tomaka, 1996). It states that people perform worse on complex tasks and better on simple tasks when in the presence of others because of the type of cardio-vascular response to the two types of tasks (Blascovich and Tomaka, 1996). When performing a simple task in the presence of others, people show a normal cardio-vascular response. However, when performing a complex task in the presence of others, the cardio-vascular response is similar to that of a person in a threatening position. The normal cardio-vascular response serves to improve performance, but the threat-like cardiovascular response serves to impede performance. Consequences for suboptimal performance include not only financial losses but also poor evaluations by others.

However, recent laboratory experiments with college students and experiments in rural India demonstrated that arousal associated with high reward levels can have detrimental effects on performance across diverse skill domains (Ariely et al., 2009). These results speak to a general detrimental role of arousal on tasks in general irrespective of the nature of the task and the level of skills. Further, compared with the two attentional models mentioned above, the mechanism by which high arousal influences behaviors is relatively unclear in the over-arousal model.

## The neural basis of choking under pressure

All these accounts of choking, the distraction, explicit monitoring, and over-arousal accounts, are not mutually exclusive but complement each other. However, although behavioral studies are useful in teasing apart these hypotheses, they are silent with respect to the neural underpinnings of choking and possible biological mechanisms. Recent advances in functional neuroimaging have allowed researchers to examine brain activity *in vivo* when participants are doing experimental tasks. Accumulating evidence suggests that certain brain circuits are specifically engaged in attentional control, performance monitoring, emotion regulation, reward processing, and motivation. Thus, human neuroimaging studies may provide neural evidence to support and/or dissociate competing models of choking under pressure. The dorsolateral prefrontal cortex (DLPFC) and the anterior cingulate cortex (ACC) have been implicated in error detection (Swick and Turken, 2002; Koban and Pourtois, 2014), conflict monitoring (Mansouri et al., 2009; Iannaccone et al., 2015), and emotion regulation (Shackman et al., 2011; Frank et al., 2014). In addition, reward is fundamental to emotion, motivation, learning, and goal directed behavior, and it is not surprising that our brain has specific circuits devoted to processing rewards in an efficient manner, providing a critical evolutionary advantage for survival. Electrophysiological studies in non-human primates have established that single-cell firing rates are modulated by reward within the meso-limbic-cortical pathway, from the ventral tegmental area (VTA) via striatum to the medial prefrontal cortex (Pierce and Kumaresan, 2006). Brain responses in these reward sensitive regions have been elicited by primary rewards, such as juice and water, as well as a number of secondary rewards such as money, beautiful faces, humor, and other social rewards, suggesting that the brain may process rewards along a single common pathway (Kim et al., 2011; Lin et al., 2012; Sescousse et al., 2013, 2014).

Each of these theories of choking would make different predictions about brain activity. The distraction and explicit-monitoring theories would predict that activity in executive-control-related brain regions would be associated with choking under pressure. Specifically, if the distraction theory is correct, one would predict that activity in attention control regions would be reduced when individuals choke and the diminished engagement of attention control regions would predict the degree of choking. On the other hand, in favor of the explicit monitoring theory, results would show enhanced activity in attention control regions and this over-activity would be associated with individuals’ propensity to choke. The over-arousal theory would hypothesize the involvement of reward sensitive regions in choking effects.

### Task-based fMRI

In a functional magnetic resonance imaging (fMRI) study, Mobbs et al. developed a Pac-Man like game in which participants controlled a blue triangle (the predator) to capture an artificially intelligent prey in order to win either a low (£0.5) or a high reward (£5) (Mobbs et al., 2009). The behavioral results revealed that participants were less successful in catching the high-payoff prey than in catching the low-payoff prey and made more erroneous actions in the high- than in the low-reward condition. Neuroimaging data showed that as participants were closer to the prey, activity in reward regions escalated, including dorsolateral striatum, ventral striatum, medial orbitofrontal cortex, and rostral ACC. Interestingly, the parametric effect of distance in the left ventral midbrain, encompassing the VTA/substantia nigra, right dorsal striatum, and bilateral ventral premotor area was significantly stronger in high-reward vs. low-reward conditions, suggesting that these regions encode reward incentive as a function of goal distance. Midbrain activity was also correlated with performance decrements and increases in errors. Activities in the right ACC and medial prefrontal cortex showed the opposite patterns and were correlated with better performance and reduced susceptibility to incentive-induced errors in the high-payoff condition relative to the low-payoff condition. These findings suggest that excessive motivation registered in the midbrain produces choking, whereas increased cortical control may reduce it, supporting the over-arousal theory. Because the midbrain is sufficiently rich in dopaminergic cells and is the key part of the mesocorticolimbic dopamine system, these results also imply a potential role of the neurotransmitter dopamine in choking under pressure. Consistent with this speculation, a positron emission tomopraphy (PET) study found that tonic dopamine synthesis was positively associated with performance decrements in a cognitive control task (i.e., the Stroop task) when incentives were high (Aarts et al., 2014), suggesting that monetary bonuses may impair cognitive control via over-exciting the dopaminergic system (Silston and Mobbs, 2014).

Another fMRI study showed that when actually performing a motor task, individuals encoded the potential loss that would arise from failure (Chib et al., 2012). Between initial incentive presentation and task execution, striatal activity rapidly switched between activation and deactivation in response to increasing incentives, regardless of whether trials were unsuccessful or successful. Decrements in performance and striatal deactivations were directly predicted by an independent measure of behavioral loss aversion in 12 subjects. Follow-up behavioral assessments further showed that behavioral loss aversion was correlated with performance decrements in high reward contexts in 32 subjects, strengthening the link between loss aversion and choking. This study highlights the role of loss aversion in choking under pressure. Nevertheless, it is still difficult to disentangle the excitement of possible success (e.g., reward sensitivity) and the fear of loss due to the intimate relationship between the two. The link between loss aversion and susceptibility to choking effects was replicated in a later study in which incentives were either framed as gain (to obtain potential gains) or loss (to avoid losing money) (Chib et al., 2014). Interestingly, the striatum was activated for both increasing prospective gains and increasing prospective losses at the time of incentive presentation. These findings provide neural evidence to support the over-arousal theory. A recent fMRI using a challenging visuomotor task (similar to the classic arcade video game *Snake*), however, found no correlation between loss aversion and decrements in performance under high incentive (*p* > 0.5) (Lee and Grafton, 2015). Instead, there was a significant positive relationship between trait impulsivity and choking. Moreover, it was found that the functional connectivity between DLPFC and motor cortex was inversely related to the propensity to choke under pressure (Lee and Grafton, 2015). This study emphasized the role of prefrontal cortex control regions rather than the midbrain/striatum motivation regions in choking. The negative correlation between frontal-motor functional connectivity and choking is consistent with a distraction account of choking (Lee and Grafton, 2015), suggesting that choking is due to the lack of executive control resources in frontal regions.

Some students crack under the stress of important math tests in which the desire to perform their best is extremely high. For these individuals, being faced with the prospect of doing math itself can evoke intense anxiety, which mitigates math-specific performance deficits. These highly math anxious (HMAs) individuals can be identified via a common self-report measure of math-anxiety, the Short Math-Anxiety Rating Scale. Using fMRI, Lyons and Beilock examined neural activity in subjects while they were anticipating doing math and when performing the task itself (Lyons and Beilock, 2012a). When simply anticipating doing math, increased activity in frontoparietal network (e.g., bilateral inferior frontal junction) was observed for higher math-anxious individuals. The inferior frontal junction is involved in reappraisal of negative emotional responses. Furthermore, the relation between frontoparietal anticipatory activity and highly math-anxious individuals’ math deficits was fully mediated by activity in caudate, nucleus accumbens, and hippocampus during math performance. These subcortical regions are implicated in motivation. Further analysis shows that individual differences in how math anxious individuals recruit cognitive control resources prior to doing math and motivational resources during math performance predict the extent of their math deficits. In this study, a self-report measure of math anxiety was not correlated with math deficits in the HMA group, showing that it is not poor performance that causes mathematics anxiety. Overall, these results indicate that both attention distraction and over-arousal contribute to choking and the interaction between the two processes may be crucial in understanding the choking phenomenon in math performance. Another study from the same group found that anticipating an upcoming math task activated the pain network, consisting of the mid-cingulate cortex (MCC) and insula, in individuals reporting high math anxiety (Lyons and Beilock, 2012b). This result suggests that simply being faced with the prospect of doing math is psychologically painful. This finding also supports the over-arousal (over-anxious) view of choking.

These studies, using either cognitive or sensorimotor tasks, have demonstrated that brain regions that are important for motivation and emotion regulation are involved in choking under pressure. Differential brain responses during the anticipation stage and execution stage therefore highlights the need to dissociate these two phases. Depending on the source of psychological pressure, distinct brain regions are engaged during the anticipation stage. If the pressure comes from high monetary reward, brain regions that register potential reward are recruited, possibly signaling the excitement of obtaining potential reward (Mobbs et al., 2009; Chib et al., 2012). When the pressure is provoked by the fear of failure, brain areas implicated in encoding negative valence are activated (Lyons and Beilock, 2012b). However, it is also possible that the striatum is encoding the level of general arousal elicited by either excitement of prospective reward or fear of losing (Chib et al., 2014). Of importance, stress may not only influence performance but also affect the encoding of incentives itself. A number of studies have shown that incentive-irrelevant stress, induced by a cold pressor or negative social feedback, increased striatal and amygdalar activation during anticipation of reward (van den Bos et al., 2009; Mather and Lighthall, 2012; Porcelli et al., 2012; Starcke and Brand, 2012; Kumar et al., 2014; Lewis et al., 2014). It is possible that high incentive elicits stress and stress in turn amplifies the sensitivity to incentives. During task execution, studies revealed diminished activity in the motivation regions (Chib et al., 2014) and in prefrontal-parietal networks (Lyons and Beilock, 2012a; Lee and Grafton, 2015) in the stressful conditions. Taken together, these findings suggest that over-arousal and diminished executive control both may contribute to choking under pressure. However, the functional significance of these brain activity patterns in response to stress is worthy of further examination because these explanations are generated in part from a “reverse-inference” (Poldrack, 2006).

### Stress-state fMRI

Another line of research has examined neural responses as well as functional connectivity between brain regions during the stressful state in heathy individuals. In a study using the Montreal Imaging Stress Task (MIST), a social stress paradigm in which participants solve arithmetic tasks under time pressure, significant deactivation of the limbic system including hippocampus, hypothalamus, medio-orbitofrontal cortex and ACC was observed in subjects who reacted to the stressor with increased cortisol (Pruessner et al., 2008). Moreover, the degree of deactivation in the hippocampus was correlated with the release of cortisol in response to the stress task. Urban living is linked to a more demanding and stressful social environment. In another study using the MIST, researchers examined the neural activity under stress in both city dwellers and rural dwellers (Lederbogen et al., 2011). Across groups, stress-related brain activations were identified in the right temporoparietal junction, ACC and posterior cingulate cortex, insular cortex and hypothalamus. Differential brain activation correlated significantly with the test-induced rise in cortisol for hippocampus and amygdala, replicating the previous study. Importantly, current urban living was associated with amygdala activity in response to stress, and urban living in the early years was positively correlated with activity in pregenual anterior cingulate cortex (pACC), a key region involved in regulation of amygdala activity and stress (Diorio et al., 1993), suggesting that stress associated with city life may produce neural effects.

Taken together, these studies have identified key regions that respond to stress manipulation, but are silent on the crosstalk among these regions and pathways. A recent study, employing functional connectivity methods, investigated a neural networks signature of stress. During exposure to a fear-related acute stressor, responsiveness and interconnectivity within a network including cortical (frontoinsular, dorsal ACC, inferotemporal, and temporoparietal) and subcortical (amygdala, thalamus, hypothalamus, and midbrain) regions increased as a function of stress response magnitudes (Hermans et al., 2011). Previous studies have shown that these regions are associated with interoception (Wager et al., 2009a,b), autonomic-neuroendocrine control (Schwabe et al., 2012), catecholaminergic singaling (Ulrich-Lai and Herman, 2009), attentional orientating (Corbetta et al., 2008), and salience processing (Seeley et al., 2007; Uddin, 2015). These findings suggest that stress promotes information exchange between regions involved in autonomic-neuroendocrine control and vigilant attentional reorienting, which may foster rapid defense mechanisms (Hermans et al., 2011). Importantly, b-adrenergic receptor blockade, but not cortisol synthesis inhibition, diminished such increase. This finding suggests that neuromodulator noradrenaline drives this network reorganization. In a recent perspective paper, these researchers discussed the possible involvement of two brain networks, the salience (e.g., emotional reactivity and attentional vigilance) vs. executive control network (e.g., working memory and decision making), in governing stress (Hermans et al., 2014). Hermans et al. implied that the salience network, which includes anterior insula, midbrain, thalamus, and dorsal ACC, may be more associated with sympathetico-adrenal (epinephrine and norepinephrine) stress axis (Hermans et al., 2014). The fronto-parietal executive control network might be more related with the hypothalamo-pituitary-adrenal (cortisol) stress axis (Hermans et al., 2014). The two stress systems may play differential roles in the neurobiology of performance under pressure. Emotional overreaction, the overactivation of sympathetic nervous system as reflected by sweaty palms, rapid heartbeat, and so on, may worsen performance under pressure, whereas activation of executive control networks could improve performance under pressure. It is plausible that a balanced activation of the salience network vs. execute control network is needed for proper performance under pressure (Hermans et al., 2014).

Although this dual-system model remains to be tested, the findings that choking was associated with exaggerated activity in motivation-related regions such as midbrain, striatum, and insula (Mobbs et al., 2009; Chib et al., 2012, 2014; Lyons and Beilock, 2012b) and diminished prefrontal activity (Lyons and Beilock, 2012a; Lee and Grafton, 2015), support the dual-system view of stress and performance. Thus, there exists a consistency between findings from task-based neuroimaging and results generated by large-scale neurocognitive network analysis. Both approaches demonstrate that activity in cortical regions is weakened and neural responses in subcortical brain areas are amplified when individuals are under stress. Although the stress-state-related fMRI studies involve no decreased behavioral performance or individuals who are prone to choking, it is tempting to predict that the identified neural network coupling under stress might be associated with choking. It remains to be tested what differentiates “chokers” from those who thrive under stress in terms of brain network configuration. Given the complexity of the question, in addition to behavioral and neuroimaging research, a multidisciplinary approach involving pharmacological manipulations, brain stimulation methods should be employed, in order to establish causal relationships.

### Limitations in neuroimaging studies on choking

Several caveats need to be mentioned for the neuroimaging approach. First, it is worth noting that the interpretation of neuroimaging results in these previous studies is, in essence, a form of reverse inference (Poldrack, 2006). For example, the ACC, insula, and amygdala are also engaged in saliency detection and may not encode stress or negative emotion *per se* in those experimental tasks. Second, these studies are correlational and any causal conclusion should be drawn cautiously. Future avenues of research toward establishing causal relationships between activities in certain brain regions and choking may employ brain stimulation, pharmacological manipulation, and lesion studies. Third, it is worth noting that high incentives do not always lead to high motivation to work. Behavioral studies have also shown that high incentives reduce labor input (Sanders and Walia, 2012). It is possible that individuals change the way they evaluate prospective rewards or losses when they are over-stressed by those prospects. For example, they may downregulate the importance of the reward. Fourth, unlike many stress-related studies, most fMRI studies on choking under pressure do not measure any “stress hormone” such as cortisol. Lack of physiological measurement limits the understanding of individual differences in response to stress and how brain activity is related to neuroendocrine reactions. It has been found that individuals with different coping styles (e.g., pro-active or reactive) exhibited different stress responses in the TSST and decision making patterns (van den Bos et al., 2013b). The three dominant theories of choking also do not point to the role of stress hormone in the choking processes. Future empirical research should devote more efforts to delineate the relationship between choking and stress hormones. Finally, the underlying mechanisms for choking remain to be further illustrated. It seems to be circular to argue that over-motivation, loss aversion, or worries about tests contribute to choking. Why and how over-motivation and loss aversion cause performance deficits is still an open question. The specific cognitive processes and the associated neural activity during choking need to be addressed in future research. Attentional theories offer a detailed explanation of the processes and the neural correlates of these cognitive processes waiting to be identified. Specifically, distraction or monitoring theories would predict different types of attention allocation and working memory deployment during task implementation. The interaction between motivation network and cognition network in the human brain will be an important topic in future exploration of the choking phenomenon.

## Combating choking under pressure

Based on our knowledge of the precursors of choking, interventions for performance under pressure can be developed to combat choking in high-stakes situations. Several recent studies have already directly examined how a variety of interventions can mitigate the choking effect. These interventions in turn can also increase our understanding of why pressure-filled exam situations undermine some students’ performance.

If the ability of working memory to maintain task focus is disrupted because of situation-related worries, performance can suffer. Anxiety and worries compete for the working memory available for performance. Before anxiety has a chance to reduce actual performance, eliminating one’s initial anxiety response at the early stage seems to be important. Expressive writing, in which people repeatedly write about a traumatic or emotional experience in the past, has been shown to be effective in decreasing rumination in depressed individuals (Smyth, 1998). Writing may alleviate the burden that worries place on working memory by affording people an opportunity to reevaluate the stressful experience in a manner that reduces the necessity to worry altogether. In two laboratory and two randomized field experiments, a recent study found that having students write down their thoughts about an upcoming test could improve test performance (Ramirez and Beilock, 2011). The intervention, a brief expressive writing assignment that occurred immediately before taking an important test, significantly improved students’ exam scores, especially for high math-anxious students. This study indicates that simply writing about one’s worries before a high-stakes event can boost performance. Whether this simple technique is also effective in other stressful situations awaits further test. The success of this intervention further corroborates the over-arousal theory of choking.

Negative thoughts and worries can also be curtailed by reappraisal or re-framing techniques. Reappraising the situation or the meaning of their anxiety provides threatened individuals a means to effectively cope with negative emotions. Reappraisal, particularly distraction, has been shown to alleviate choking under pressure (Balk et al., 2013). Re-framing metacognitive interpretation of difficulty, e.g., simply telling students that physiological responses (e.g., sweaty palms, rapid heartbeat) are beneficial for thinking and reasoning, can improve test performance in stressful situations (Autin and Croizet, 2012). Intuitively, any interventions that can keep pressure-induced worries at bay may also help in alleviating pressure-induced choking. However, it is important to note that emotion regulation during tasks may backfire because regulation also depletes executive resources needed to perform well. The fear extinction techniques used to diminish conditioned fear or phobias may also be considered in future studies (Monfils et al., 2009; Quirk et al., 2010). Traumatic choking under pressure experience may create a high-stakes situation and failure association. When in the stressful situation, the conditioned fear of failure is elicited and thus history repeats itself, creating a stress-failure cycle. The new stress-success link, if established, may overwrite the previously learned association and thus break the choking cycle.

## Concluding remarks

Why do people choke and what can we do about it? The sources of pressure, the nature of the tasks, gender, and individual differences in personality and cognitive abilities, are all important determinants of performance success or failure. For example, FNE is an important psychological characteristic of the choking-susceptible athlete. The social element may make it a special source of pressure that may provoke choking via mechanisms that are different from those for monetary reward-induced choking. It has been demonstrated that stress has a different effect on cortisol responses and decision making in men and women (van den Bos et al., 2009, 2013a,b; de Visser et al., 2010). Moreover, how incentive-induced pressure differs from talk-irrelevant stress induced by ice-cold water or in the TSST is also an interesting topic for future studies (Starcke and Brand, 2012; van den Bos et al., 2013b). While sources of stress have indeed been investigated independently, they have not been systematically studied and compared yet. There might be multiple routes to performance failure, engaging distinct brain networks. Moreover, in addition to examining individuals who choke, future research may also pay attention to those who thrive under stress and adversity, which is largely neglected in both the theoretical and empirical research literature on stress. Identifying determinates of such resilience to stress can also aid in engineering training regimens that better equip individuals to meet the challenges in the modern society (Coppens et al., 2010; Koolhaas et al., 2010; van den Bos et al., 2013b). Understanding choking is crucial for psychological theories of motivation and it also carries empirical implications for research in sports, business, and management. Understanding the complex and nonlinear relationship between performance-contingent incentives and actual performance can help business agents design better payoff schemes to incentivize workers. However, although societies are responsible to offer optimal incentives to motivate but not to over-motivate employees, it is impossible to create a stress-free world for citizens. Humans inevitably have to perform in situations when the stakes are extremely high, for example in emergency rooms (LeBlanc, 2009). Thus, enhancing individuals’ resistance to choking is an impressing issue. Behavioral interventions, such as expressive writing and reappraisal, aiming at reducing arousal and anxiety levels, have been shown to be successful in combating choking. Other interventions that directly deal with attention allocation and cognitive control may also help reduce pressure-induced failure and these techniques need to be explored. Neuroimaging findings may guide contemporary neuroscience methods including neurostimulation and psychopharmacology to target the key regions and neurotransmitters that are involved in choking under pressure. For instance, neurostimulation such as transcranial direct current stimulation (tDCS) can also be utilized to stimulate the area of interest, which may enhance individuals’ ability to control motivation and attention. A better understanding of what governs crashing under pressure may aid individuals to thrive but not choke in the most important moments in life.

## Conflict of interest statement

The author declares that the research was conducted in the absence of any commercial or financial relationships that could be construed as a potential conflict of interest.
